# Dental anxiety: detection and management

**DOI:** 10.1590/S1678-77572010000200001

**Published:** 2010

**Authors:** Mohammad O. SHARIF

**Affiliations:** BDS (Hons), MJDF RCS Eng, National Institute for Health Research In-Practice Research Training Fellow. School of Dentistry, University of Manchester, Manchester, United Kingdom.

Historically dental anxiety has been attributed to the expectation of pain^1,4,10^. Over the past century this has been the main driver for improvements in pain control. However, despite the advances in pain control worldwide figures on the prevalence of dental anxiety are still in the region of 10-15%^2,7,14^ and therefore it is still a significant barrier to dental care for a consistent proportion of the population^5,9^. Dental anxiety not only leads to the avoidance of dental care but it also effects individuals generally, one report has shown that it causes sleep disturbance, negative thoughts and feelings of low self esteem and confidence^3^.

## Detection of dental anxiety

In a large number of cases clinical impression alone will alert the clinician of the presence of anxiety. Subjective assessment can be used as well as formalized questionnaires^8^. One example of the use of formalized questionnaire used for adults is the Modified Dental Anxiety Scale (MDAS), this is a brief five item questionnaire, which is used to help objectively identify patient anxiety levels^6^.

For children picture tests such as the ‘Venham Picture Test’ are commonly used, the child indicates his/her level of anxiety by picking out a picture that illustrates their perceived emotion^7^. The images commonly used are faces with a value of 1-5 with 5 representing higher dental fear.

## Management of dental anxiety

**Figure 1:**
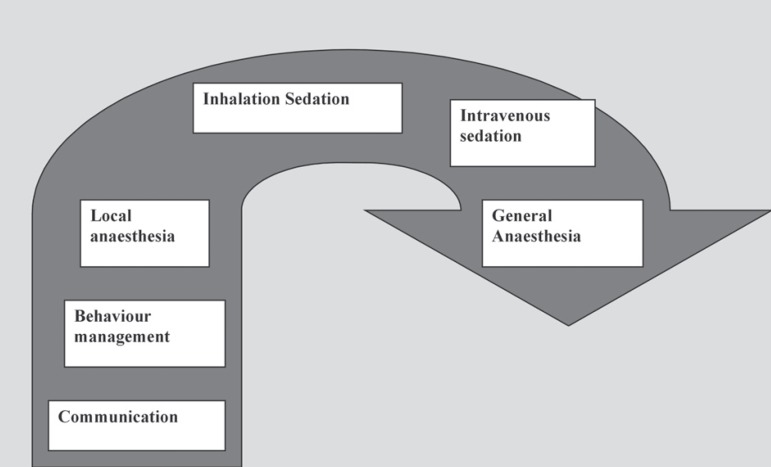
Spectrum of anxiety management approaches

Approaches for dental anxiety management should be discussed with the patient. This will provide the patient with a feeling of involvement and helps them cope with the stresses associated with dental visits more effectively^11-13^. For anxiety management to be effective it should be tailored for individual patients. There is spectrum of options that should be employed for anxiety control in which least invasive approaches are used first i.e. non-pharmacological approaches (communication, behaviour management) and local anaesthesia (pain control). Should these fail to control anxiety effectively or it is anticipated that these approaches will be insufficient, we then move onto the use of pharmacological adjuncts (inhalation sedation, intravenous sedation and general anaesthesia) (Figure 1).

## Conclusion

The effective management of dental anxiety is of paramount importance, this management needs to consist of a multifaceted approach. For the approach to be effectively tailored to provide maximum benefit for patients dentists need to be efficient at detecting the presence of anxiety and be able to tailor management according to patient needs.
